# Silver Nanoparticles in Dental Applications: A Descriptive Review

**DOI:** 10.3390/bioengineering10030327

**Published:** 2023-03-05

**Authors:** Sreekanth Kumar Mallineni, Srinivasulu Sakhamuri, Sree Lalita Kotha, Abdul Rahman Gharamah M. AlAsmari, Galiah Husam AlJefri, Fatmah Nasser Almotawah, Sahana Mallineni, Rishitha Sajja

**Affiliations:** 1 Pediatric Dentistry, Dr. Sulaiman Al Habib Hospital, Ar Rayyan, Riyadh 14212, Saudi Arabia; 2 Division for Globalization Initiative, Liaison Center for Innovative Dentistry, Graduate School of Dentistry, Tohoku University, Sendai 980-8575, Japan; 3 Center for Transdisciplinary Research (CFTR), Saveetha Institute of Medical and Technical Sciences, Saveetha Dental College, Saveetha University, Chennai 600077, Tamil Nadu, India; 4 Department of Conservative Dentistry & Endodontics, Narayana Dental College and Hospital, Nellore 523004, Andhra Pradesh, India; 5 Department of Basic Dental Sciences, College of Dentistry, Princess Nourah bint Abdulrahman University, P.O. Box 84428, Riyadh 11671, Saudi Arabia; 6 Ministry of Health, Al Qunfudah Dental Center, Al Qunfudah 28821, Saudi Arabia; 7 Preventive Department, Dar al Uloom University, Riyadh 13314, Saudi Arabia; 8 Preventive Dentistry Department, Pediatric Dentistry Division, College of Dentistry, Riyadh Elm University, Riyadh 13244, Saudi Arabia; 9 Department of Periodontology, Krishna Institute of Medical Sciences, Nellore 523001, Andhra Pradesh, India; 10 Clinical Data Management, Global Data Management and Centralized Monitoring, Global Development Operations, Bristol Myers Squibb, Pennington, NJ 07922, USA

**Keywords:** dentistry, nanotechnology, silver nanoparticles, AgNPs, antimicrobials

## Abstract

Silver nanoparticles have been a recent focus of many researchers in dentistry, and their potential uses and benefits have drawn attention in dentistry and medicine. The fabrication and utilization of nanoscale substances and structures are at the core of the rapidly developing areas of nanotechnology. They are often used in the dental industry because they prevent bacteria from making nanoparticles, oxides, and biofilms. They also stop the metabolism of bacteria. Silver nanoparticles (AgNPs) are a type of zero-dimensional material with different shapes. Dentistry has to keep up with changing patient needs and new technology. Silver nanoparticles (AgNPs) can be used in dentistry for disinfection and preventing infections in the oral cavity. One of the most interesting metallic nanoparticles used in biomedical applications is silver nanoparticles (AgNPs). The dental field has found promising uses for silver nanoparticles (AgNPs) in the elimination of plaque and tartar, as well as the elimination of bacterial and fungal infections in the mouth. The incorporation of AgNPs into dental materials has been shown to significantly enhance patients’ oral health, leading to their widespread use. This review focuses on AgNP synthesis, chemical properties, biocompatibility, uses in various dental fields, and biomaterials used in dentistry. With an emphasis on aspects related to the inclusion of silver nanoparticles, this descriptive review paper also intends to address the recent developments of AgNPs in dentistry.

## 1. Introduction

**Figure 1:**
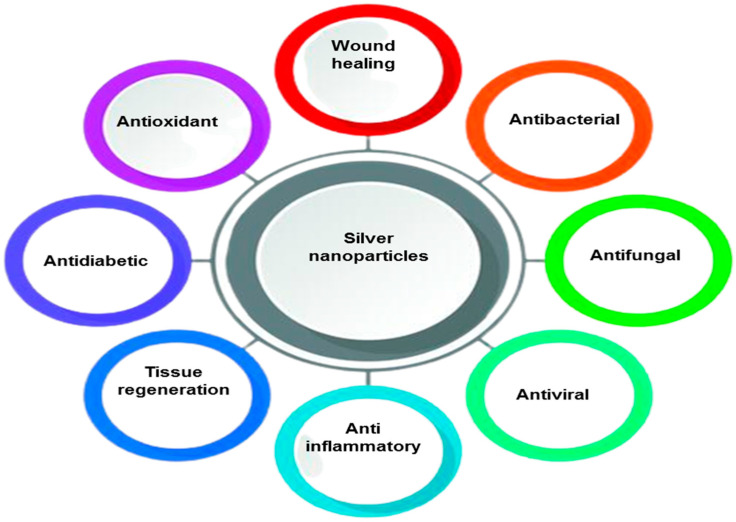
Significance of silver nanoparticles.

Silver (Ag) ions or salts have significant antibacterial capabilities and have been employed in medical applications for years. Sterile gauze, catheterization, and prosthesis are examples of this. Ag’s low toxicity, great biocompatibility with human cells, and constant mobile ions provide long-lasting bactericidal action [1]. Silver nanoparticles (AgNPs) are widely used in medicine because they uniquely interact with numerous microbial species. Wound stitches, tracheostomy, surgical equipment, and bone prosthesis are examples of this. Endodontics, dental prostheses, implantology, and restorative dentistry use AgNPs. AgNPs may lower bacterial populations in dental composites, improving oral health and overall well-being [2]. Due to their small size, gNPs exhibit different chemical, physical, and biological properties to bulk materials. They are effective antibacterial fillers because of their small size and vast surface area [3]. The significance and properties of silver nanoparticles are shown in Figure 1.

The term “Nano” derives from the Greek word “Nano”, which signifies dwarf or extremely little [4]. The phrase refers to particles comprising between 20 and 15,000 Ag atoms and ranging in size from 1 nm to 100 nm [5]. AgNPs’ antibacterial, antifungal, and antiplasmodial activities have been well known in the biomedical sector for decades [4]. Nonetheless, the antibacterial impact is affected by the concentration and velocity of ionic Ag emission. AgNPs may directly interact with bacteria, modify their basic makeup, and cause harm cells due to their nanoscale size and relatively large external regions [5]. Nanoparticles of various sizes and shapes can be created using a variety of processes, including physical, chemical, and biological synthesis [6]. Streptococcus mutants, the main bacterium responsible for dental cavities, have been shown to be resistant to AgNPs’ AB and anti-adherence properties. AgNPs with diameters ranging from 1 to 10 nm have strong antibacterial and anti-adherence effects against “*S. mutans* bacterium” [7]. There are several bacterial species, each with their own membrane structure. Gram-positive bacteria (GPB) and Gram-negative bacteria (GNB) are two often-used classifications. The density of the cell wall and the presence of an outside lipid membrane are the two key characteristics that distinguish the two. GPB lacks an external lipid membrane, but its cell walls are formed of a peptidoglycan layer about 30 nm thick. GNB microorganisms have a lipopolysaccharide membrane surrounded by a visible peptidoglycan layer (2–3 nm) [8].

**Figure 2:**
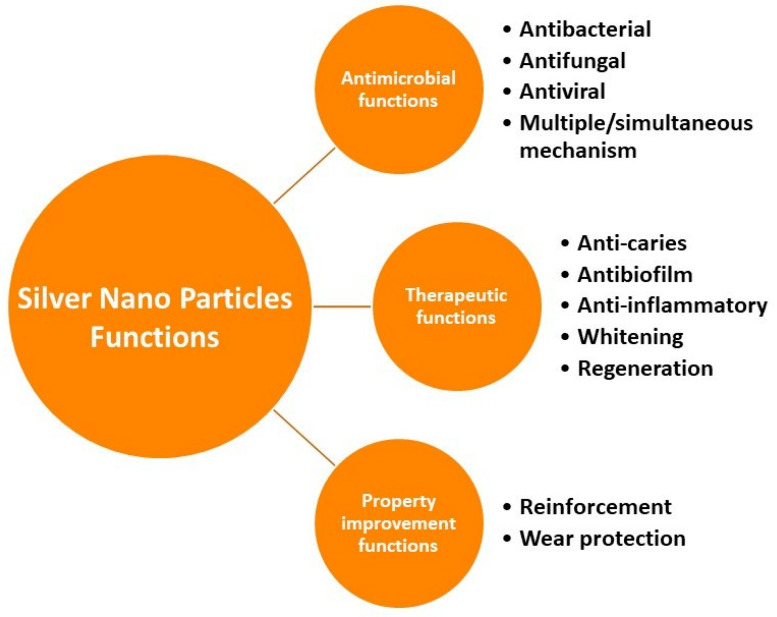
Functions of silver nanoparticles.

Based on their functions, silver nanoparticles have been used in a variety of healthcare disciplines. Silver nanoparticles have functions [9] that include antibacterial, medicinal, and property improvement actions (Figure 2).The published literature focuses on the incorporation of silver nanoparticles (AgNPs) into various dental materials, such as composite resin and adhesive systems, acrylic resin, root canal fillers, and implantable devices, with special attention paid to the inhibition of microbial growth, neurotoxic effects, and altered physical properties of the materials discussed in this section. Several writers have investigated and reviewed the use of silver nanoparticles in dentistry, and there is still a need to discuss updates on the usage of AgNPs in dentistry. This review focuses on several elements of dentistry, such as the dental implications of nanotechnology, clinical uses of nanoparticles in dentistry, and dental biomaterials, particularly the effect of including silver nanoparticles (AgNPs).

## 2. Silver Nanoparticle Chemistry and Synthesis

Metallic nanoparticles (NPs) are typically produced using chemical synthesis processes that reduce the metal salts of NPs and disperse them in an organic or aqueous solution. A number of metallic salts are used to make gold, silver, iron, zinc oxide, copper, palladium, platinum, and other analogous metal nanospheres. The nucleation and formation of NPs are controlled by several reaction parameters, such as reaction temperature, pH, concentration, type of precursor, reducing and stabilizing agents, and the molar ratio of surfactant/stabilizer and precursor. Different chemical reductants, such as glucose (C_6_H_12_O_6_), hydrazine (N_2_H_4_), hydrazine hydrate, ascorbate (C_6_H_7_NaO_6_), ethylene glycol (C_2_H_6_O_2_), N-dimethylformamide (DMF), hydrogen, dextrose, ascorbate, citrate (Turkevich method), and sodium borohydride, can be used to chemically reduce these metal salts (BSS method) [10]. Brust and colleagues invented the most widely used method for producing thiol-stabilized AuNPs and AgNPs. In an aqueous solution, the silver ion (Ag+) undergoes a reduction, transitioning from a positive valence to a zero-valent state (Ag0), followed by nucleation and growth. As a result of coarse agglomeration into oligomeric clusters, colloidal AgNPs are formed [11]. Previously, small monodispersed colloids were generated by employing a strong reductant (borohydride) but regulating the development of larger AgNPs proved problematic. Using a weaker reductant, such as citrate, resulted in a slower reduction rate, which was more suitable for managing the shape and size distribution of NPs. Silver nanoparticles are produced using a variety of methods, including physical, chemical, and biological synthesis. It is critical to note that each method has advantages and disadvantages. During the biological production of silver nanoparticles, the organism reduces Ag+ to generate Ago, functioning as a capping agent, reducing agent, or stabilizing agent [11]. Because of their low cost, high yields, and low toxicity to the environment and human body, biological technology based on natural materials derived from plant and microbial sources have witnessed a surge in use in recent years [12]. Table 1 summarizes silver nanoparticle synthesis approaches for use in dentistry [13,14,15,16].

## 3. Dental Implications of Nanotechnology

### 3.1. Ethics and Implications

After considerable research and development, dental and medical nanoproducts undergo preclinical in vitro testing to assess their mechanical, toxicological, and immunological properties. Many authorities, such as the National Institute of Occupational Safety and Health and the US Environmental Protection Agency, have nanoparticle risk guidelines [17]. The ethical committee’s utilitarian decision-making process cannot keep up with nanotechnology discoveries and the products’ rapid expansion and uncertain future. Therefore, risk/benefit calculations, ethics, and a broader grasp of science are needed throughout the development process [18]. Anticipatory ethics and governance were advocated. Ethical analysis models were used to recognize and handle ethical and societal implications when the technology was young, allowing for it to be updated and guided toward a moral conclusion [19]. Nanoproducts must undergo in vitro mechanical, toxicological, and immunological testing before use in medicine or dentistry [6]. Labs perform these tests. Several groups have proposed nanoparticle safety research: “Legislators are still working on a multidisciplinary regulatory framework to explore and manage nanotechnology and handle ethical issues in metaphysics, equality, privacy, and security. [7]. Even while animal studies provide ample knowledge of what to expect before starting a phase I trial, caution should still be maintained because study subjects who were administered nanoparticles 500 times lower than the toxic level in animal research have had serious adverse effects”. Data and safety monitoring committees and warnings about novel items must be given to clinical trial participants [8]. These boards monitor and record unpleasant side effects, detect data handling errors, and ensure the test subjects’ safety and well-being [9]. The utilitarian approach to ethics cannot keep up with nanotechnology’s rapid growth and unpredictable future. Thus, anticipatory ethics and governance are necessary [20]. This idea aims to use ethical analysis models early in technology development to identify and address moral and social issues. Thus, the technology can be easily shaped to accomplish ethical results [21].

### 3.2. Society and Nanotechnology

Society is the client, financier, and policymaker. Thus, nanotechnology’s success depends on public opinion. New sparking technologies have made ethics, morality, and values more sensitive [22] because the technology’s benefits outweigh the risks. Nanotechnology is already used in energy provision, wellness and diagnostics, communication, and environmental management, but there are still challenges to overcome. There is a concern that these advancements will deprive the public of thousands of jobs to make room for a more machine-reliant system [23]. Several attempts have been made to bridge society and nanoscience to solve societal issues. The National Nanotechnology Initiative (NNI) study helps US federal agencies organize nanoscale research resources. As technology evolves, additional specialists with sophisticated training and operational and administrative skills are needed [24].

### 3.3. Implications for Health

Federal and state agencies in the US have developed a four-stage framework for examining and evaluating health issues [25]. These stages are as follows: define the problem; determine the dose–response relationship; and characterize the risk [26]. When lowered to 1 nm, harmless particles with a length of 100 nm can swiftly become harmful components [27]. When fragmented or clumped, non-poisonous nanomaterials can become hazardous nanoparticles [28]. Nanomaterials respond differently in cultured cells than in organisms, making it hard to anticipate how humans might react [29]. Science has outpaced ethical, economic, and health concerns about nanotechnology [30]. Despite funding [31], no major investigations have been carried out [32]. Instead, hype and controversies fuel public mistrust of disruptive new technology [33].

### 3.4. Biocompatibility

New biocompatible dental materials have been developed to protect the mouth’s keratinocytes and oral mucosal tissues [34]. Biocompatible nanoparticles have low toxicity, and are digestive-system-friendly and immune-boosting [34,35]. Nanomaterials harm organs. Nanomaterials interact with functional foods and nutraceuticals [35]. Innocuous particles with a length of 100 nm can swiftly become harmful components when their size is lowered to 1 nm [36]. When fragmented or clumped, non-poisonous nanomaterials can become dangerous nanoparticles [37]. Nanomaterials respond differently in farmed cells than in organisms, making it hard to predict how humans might react [38]. Nanoscale ethics, economics, and health have lagged behind science [39]. Despite funding [40], no meaningful investigations have been carried out [41]. Instead, hype and controversies fuel public suspicion of disruptive new technology [42].

## 4. Clinical Applications of Nanoparticles in Dentistry

### 4.1. Preventive Dentistry

AgNPs improved self-etching adhesives’ antibacterial durability. *S. mutans* was significantly inhibited without affecting resin adhesiveness [43,44]. AgNP-based two-step adhesive solutions outperformed self-etchers in shear strength [45]. AgNP powder surpassed alcoholic AgNP solution in antibacterial activity and “self-etching adhesive” conversion. AgNPs with disinfectants created biocompatible, non-cytotoxic Nanocare Gold [46]. In first permanent molars, AgNP-based revolutionary sealant micro-leakage was compared to regular sealants. The traditional sealant had 30.6% micro-leakage, whereas the AgNP sealant had 33.3%. Fluorescence significantly decreased in the AgNP sealant group. FPS utilizing AgNPs differed by about 25% across groups. AgNPs lost fluorescence faster. The teeth remineralized after microbe-induced demineralization [47]. Silver nanofluoride (NSF) is cheaper than varnishes. Bacteriostatic NSF can prevent infection [48].

### 4.2. Endodontics

AgNPs boost the adhesive’s flexibility to nano-imperfections and have a short setting time compared to conventional sealers, particularly stiffness, poor solubility in extracellular space, and biochemical affinity to dental tissue. To improve intra-canal drugs, calcium hydroxide with AgNPs was examined regarding Enterococcus faecalis development [49]. It killed Enterococcus faecalis better than calcium hydroxide and chlorhexidine. AgNPs had the best effectiveness after one week and no antibacterial effect after one month. Thus, AgNPs are effective short-term antibacterial agents against Enterococcus faecalis [50,51]. Afkhami et al. found that silver nanoparticles (AgNPs) had better antibacterial and cytotoxic effects than 2.5% sodium hypochlorite [50]. The addition of sodium hydroxide and ethanol to this AgNPs-based irrigant is new. Sodium hydroxide dissolves pulp tissue and removes the smear layer, allowing for AgNPs to penetrate deeper. Ethanol decreases surface tension, allowing AgNPs to access dentin’s later canals and tubules. AgNPs killed *S. aureus* and *E. faecalis*, as well as 5.25% NaOCl. This irrigation solution can irrigate root canals because AgNPs have a deeper antibacterial effect. [52]

### 4.3. Dental Implants

Dental implants are more reliable than removable prostheses or permanent bridges for tooth replacement. Implant success depends on mechanical and biological factors. Biofilm buildup on titanium implants may cause failure despite their biocompatibility. Peri-implant infection might disturb patients. To prevent peri-implant infections, titanium surfaces need antibacterial coatings [53]. A single-step silver plasma immersion ion implantation approach deposited AgNPs on and underneath titanium. This approach suppressed *S. aureus* and *E. coli*. It also promoted osteoblast-like MG63 cell line development. This study demonstrated AgNPs’ biological action in a novel way. This matters because AgNPs’ physicochemical qualities directly affect surface cytotoxicity [54]. Biofilm bacteria cause dental cavities, periodontal disease, and abscesses. Structurally sound biofilms resist antimicrobials. Thus, antibacterial medication research without resistance is essential. Carboxymethyl cellulose and sodium alginate capped AgNPs for antibacterial and anti-biofilm properties [55]. Carboxy-methyl cellulose-capped AgNPs suppressed Gram-negative bacteria more than sodium alginate AgNPs. Because Gram-negative bacteria cause most periodontal infections, AgNPs may help treat them. Focusing on AgNP size and antibacterial activity, 5 nm AgNPs were more effective against oral anaerobic bacteria [56].

### 4.4. Orthodontics

Orthodontic patients frequently acquire white spots and cavities. Plaque builds around orthodontic brackets due to inadequate self-cleaning and acidogenic mouth flora. Oral biofilms may cause bracket caries and demineralization. AgNPs could greatly reduce bacteria adhesion to teeth and demineralization. AgNP-containing resin inhibited bacterial growth due to extended Ag+ ion release. Mechanical properties were unaffected by this strong biocompatibility [57]. Thus, cement with AgNPs may prevent orthodontic white spot lesions. Antibacterial silver nanoparticles (AgNPs) and hydroxyapatite (HA) amalgamated with an orthodontic adhesive were also suggested. AgNPs’ antibacterial properties were used in this investigation because HA’s early enamel lesions demineralize. Mixes of 1%, 5%, and 10% *w/w* were tested. AgNPs/HA of 5% *w/w* had the strongest antibacterial efficacy against different microorganisms [58]. Orthodontic adhesives containing a 5% composite (AgNPs/HA) prevent biofilm and cariogenic bacteria. Orthodontic adhesive SBS 1% AgNP/HA improved shear bond strength, 5% maintained it, and 10% decreased it, proving that orthodontic adhesives with 5% AgNPs/HA are antibacterial and mechanical [59]. The use of AgNPs in orthodontics is still developing; however, there is a lot of potential. AgNPs are antimicrobial and could be employed as nano-vectors for gene transfer to enhance mandibular development [60]. Research shows how such technology can be employed [60,61]. These clinical applications could increase treatment quality and minimize orthodontic costs.

### 4.5. Pediatric Dentistry

Pediatric dentistry uses AgNPs widely and AgNPs are blended with diverse materials to take advantage of their antimicrobial and smaller particle size, which decreases biofilm generation and improves oral health. Research examining AgNPs in composite resins and adhesive systems is lacking [61]. AgNPs affect the mechanical and bactericidal properties of calcium silicate blocks of cement coupled with zirconium oxides, such as mineral trioxide aggregate (MTA) and Portland cement (PC) (ZrO_2_). Compression resistance was high for PC/ZrO2 + AgNPs. AgNPs’ porosity-reducing integration may be to blame [62]. MTA and AgNPs increased mechanical properties, as did PC/ZrO2 and AgNPs. Both treatments reduced bacteria more after 15 h [63]. Thus, AgNPs improved calcium silicate cement. Composite resins and adhesives with AgNPs minimize biofilm and tooth loss.

### 4.6. Anticancer Treatment

Oral squamous-cell carcinomas are a major cause of employment disability, as well as catastrophic cosmetic problems in the mouth and face. Both radiotherapy and chemotherapy have the potential to alter homeostasis in a variety of ways. The same is true for the disadvantages of anticancer drugs, which require research on other therapeutic options. Although nanoparticles of various types have been used in medicine and dentistry, their efficiency in treating malignant tumors has yet to be demonstrated. AgNPs are cytotoxic because they can damage DNA and cause apoptosis. However, studies on the efficacy of AgNPs in cancer treatment are scarce. AgNPs reduce the development of a cancer cell line at low doses [64]. Berberine, an all-natural chemical, suppresses cancer cell development. These findings imply that AgNPs plus berberine could form an effective anticancer treatment combination. The creation of AgNPs was explored from several perspectives. Surface changes enable malignant cells to be targeted specifically [64]. These molecules can also detect cancer early, providing clinicians with a valuable tool. Because it specifically kills cancer cells while preserving healthy ones, nanocarriers reduce the common side effects of chemotherapy.

## 5. Dental Biomaterials: Effect of Silver Nanoparticles (AgNPs)

**Figure 3:**
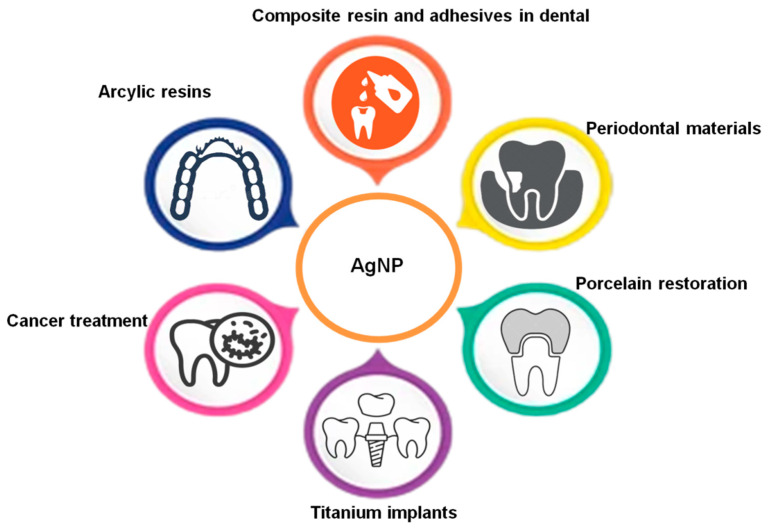
The use of AgNPs in dental bio materials.

Silver nanoparticles have been used in various dental materials and various uses of AgNPs in dental biomaterials are illustrated in Figure 3.

### 5.1. Denture Acrylic Resin

Acrylic polymers derived from poly (methyl methacrylate), also known as PMMA, are utilized in the production of partial and complete dentures used to replace missing teeth. These rough-surfaced resins are more likely to attract potentially dangerous microbes. C. Albicans, one of the most important opportunistic infections, can colonize these resins [65]. Many denture cleansers and mouthwashes have been used to eradicate these microbes [66]. These agents are incapable of eliminating these infections. The traditional resin group with no AgNPs had the maximum impact strength, whereas the microwave resin group with 0.8 weight percent AgNPs had the lowest. The addition of AgNPs to either resin group had no effect on the material’s impact resistance [67]. The heat polymerization group showed the highest glass transition temperatures; however, adding AgNPs resulted in a decline in glass transition temperatures for both groups.

### 5.2. Composite Resins

AgNPs are blended with diverse materials to take use of their antimicrobial properties, which minimize biofilm and promote oral health. Due to their tiny size, AgNPs can easily permeate cell membranes, causing damage and inactivity. Research examining AgNPs in composite resins and adhesive systems is lacking [68]. This prevents biofilm growth on the composite and restoration margins. Vazquez-Garcia et al. examined how AgNPs affect the mechanical and bactericidal properties of calcium silicate cement blocks coupled with zirconium oxide, such as mineral trioxide aggregate (MTA) and Portland cement (PC) (ZrO_2_). Compression resistance was high for PC/ZrO_2_ and AgNPs. AgNPs’ porosity-reducing integration may be to blame [69]. MTA and AgNPs increased mechanical properties, as did PC/ZrO2 and AgNPs. Both treatments reduced test microorganisms more after 15 h. Thus, AgNPs improved calcium silicate cement. A silver-ion composite resin may inhibit oral microorganisms such as *S. mutans*. Composite resins and adhesives with AgNPs minimize biofilm and tooth loss. Ag+ ions barely affect polymerization. AgNPs affected light-cured polymerized specimens’ production of important chemicals [70]. AgNPs affected composite resins. These elutable components were proportional to AgNPs. The 0.05% AgNP compound was an exception. Composites attract biofilm more than other restorative materials. These biofilms cause secondary caries at the restoration’s margins, causing failure. Antibacterial-adhesive systems combat this. AgNP resins inhibited *S. mutans* on their surface and at a longer distance than the QADM bonding agent [71]. Both chemicals did not influence fibroblast cytotoxicity or microtensile bonding. Thus, more research is needed to prove the efficacy of these novel compounds; they may be useful in restorative cement and caries prevention. AgNPs and QADM, including adhesives, have significant antibacterial effects against biofilms without affecting bond strength [72].

### 5.3. Endodontic Materials

Root canal materials should destroy these germs to improve tooth prognosis. Root canal fillings should be gutta-percha. Nanosilver-coated gutta-percha tests obturated teeth for microleakages. Nanosilver leaks less than gutta-percha but functions similarly. Eliminating bacteria and pulp tissue prevents canal space contamination. Disinfecting instruments may overlook root canal anatomy. These constraints allow for mechanical devices to clean and sanitize the canal with different irrigants, such as sodium hypochlorite (NaOCl), ethylene diamine tetraacetic acid (EDTA), and chlorhexidine (CHX). Several studies have advocated nanoparticle irrigation systems for root canal lavage [73]. We tested AgNP solution with sodium hypochlorite for *E. faecalis* (NaOCl). As a novel intra-canal irrigant, a solution below 5 mg/L is bactericidal, similar to 5.25% NaOCl, a microorganism-fighting AgNP-containing NaOCl-based irrigant [74]. This AgNP-based irrigant has the new addition of sodium hydroxide and ethanol. Sodium hydroxide dissolves pulp tissue and the smear layer, allowing AgNPs to penetrate deeper. Ethanol lowers surface tension, allowing for AgNPs’ later entry to dentin’s canals and tubules. AgNPs kills *S. aureus*, *E. faecalis*, and 5.25% NaOCl. Because AgNPs are more antimicrobial, this irrigation solution can irrigate root canals [74]. CHX and NaOCl evaluate antimicrobial AgNPs with varied surface charges. Positively charged AgNPs inhibit *E. faecalis* the least. They are cytocompatible at low doses [75] and demonstrate the surface charge’s antibacterial and root canal cleansing effects. MTA heals pulp, perforations, and apical roots. MTA’s broad action may not function against obligate anaerobes. MTA’s antibacterial effects on specific microbes are rarely studied. Modified MTA became more bactericidal with 1% AgNPs [76]. Candida albicans bacteria have been examined with this compound. The research shows that AgNPs-modified MTA kills bacteria and fungus better than MTA. Researchers tested white MTA’s biocompatibility in vivo using AgNPs. Both medicines were subcutaneously injected into rat connective tissues. Both groups showed similar inflammation. Thus, adding 1% AgNPs to MTA did not affect animal inflammation [77]. The combination of calcium hydroxide [CaOH] and AgNPs was found to be more effective against *E. faecalis* in root canal therapy than either were alone. Ca(OH)2 alone outperformed AgNPs. This study found that AgNPs without Ca(OH)2 killed less bacteria. Application technique, particle concentration and size, and AgNP-Ca(OH)2 antagonism may explain these results. Bactericidal irrigation solutions using AgNPs as the last irrigant were tested. As shown, AgNPs killed *E. faecalis*, as well as NaOCl. Unlike the previous study’s 5.25% concentration, NaOCl at 2.25% was as effective as AgNP’s irrigants. AgNP-containing irrigants eliminate the smear layer, improving penetration. The clinical use of uniradicular teeth was innovative. A better bacterial performance was observed. Bacteria in the root canal after treatment may lead to treatment failure, according to studies. Root canal treatment eliminates germs. AgNPs in root canal filling and intra-canal irrigation are worth studying because most endodontic materials lack antimicrobial characteristics [78]. Silver nanoparticle research is increasing. Silver nanoparticles can nucleate from synthetic chemicals. Proteins and chemical groups selectively functionalize silver nanoparticles. Furthermore, antibacterial silver nanoparticles increase clinical results. Homogeneous silver nanoparticles with a controlled size, shape, and function are versatile dental material building blocks. Silver nanoparticles improve prosthetic acrylic resins, direct restoration composite resins, irrigating solutions, and endodontic obturation materials. [79].

### 5.4. Periodontology

Oral biofilms—polymicrobial communities of bacteria and yeasts—cause gum and tooth infections. Biofilm bacteria cause cavities, periodontal disease, and abscesses. Biofilm removal prevents periodontal disease and tooth loss. Mechanical treatment with adjunctive antibacterials is possible. Structurally sound biofilms resist antimicrobials. Thus, antibacterial medication research without resistance is essential. Carboxymethyl cellulose and sodium alginate protect AgNPs from bacteria and biofilms. Carboxymethyl cellulose-capped AgNPs suppress Gram-negative bacteria more than sodium alginate AgNPs. Because Gram-negative bacteria cause most periodontal infections, AgNPs may help to treat them. AgNP size and antibacterial activity of 5 nm AgNPs were most effective against oral anaerobic microorganisms. AgNPs from banana peel extract were synthesized to kill human pathogens [80].

### 5.5. Orthodontic Adhesives and Cement

Orthodontic patients acquire more white spots and cavities. Plaque builds around orthodontic brackets due to inadequate self-cleaning and acidogenic mouth flora. Oral biofilm may cause bracket caries and demineralization. Plaque biofilm can be mechanically removed and treated with antibacterial agents and fluoride. Orthodontists utilize AgNPs to prevent bacteria from clinging to teeth and demineralizing enamel. Antibacterial orthodontic band cement was created using AgNPs. White lesion progression was monitored. Resins generate AgNPs in situ. AgNP-containing resin inhibit bacterial growth due to extended Ag+ ion release. Mechanical properties are unaffected by excellent biocompatibility. Thus, orthodontic cements with AgNPs may prevent white spot lesions. Silver nanoparticles (AgNPs) and hydroxyapatite (HA) in orthodontic adhesive have antibacterial properties. AgNPs’ antibacterial properties were used in this investigation because HA’s early enamel lesions demineralize, and 1%, 5%, and 10% *w/w* mixes were tested. The 5% *w/w* AgNPs/HA is the most antimicrobial. The 5% composite (AgNPs/HA) orthodontic adhesives prevent cariogenic bacteria and biofilm growth. Shear bond strength (SBS) of orthodontic adhesive increased by 1%, was maintained by 5% AgNP/HA, and decreased by 10%, suggesting that 5% AgNPs/HA provides appropriate antibacterial and mechanical properties for orthodontic adhesives. Although AgNPs in orthodontics are still in their infancy, there is still the possibility for growth. AgNPs can be employed as nano-vectors for gene transfer to accelerate mandibular growth due to their antibacterial qualities. Future studies should show how these technologies can be utilized. Clinical applications can increase treatment quality and minimize orthodontic care costs [81].

### 5.6. Anticancer Treatment

Squamous-cell carcinomas of the head and neck account for 6% of all cancer diagnoses. Nearly 40% of them are derived from the tongue or the floor of the mouth. Those who develop metastatic disease or advanced malignancies frequently do not fare well. Early detection and combinations of radiation, surgical treatments, and chemotherapy have not considerably lowered cancer incidence or mortality. Oral squamous-cell carcinomas are a major cause of employment disability, as well as catastrophic cosmetic problems in the mouth and face. Both radiotherapy and chemotherapy have the potential to alter homeostasis in a variety of ways. The same is true for the disadvantages of these anticancer drugs, showing the need for research on other therapeutic options. Although nanoparticles of various types have been used in medicine and dentistry, their efficiency in treating malignant tumors has yet to be demonstrated. AgNPs are cytotoxic because they can damage DNA and cause apoptosis. They may also have an effect on healthy cells. However, there is a scarcity of studies on the efficacy of AgNPs in cancer treatment. AgNPs reduce the development of cancer cell lines at low doses. Berberine, an all-natural chemical, reduces cell proliferation. These findings imply that AgNPs plus berberine could form an effective anticancer treatment combination. The creation of AgNPs has also been explored from several perspectives. Surface changes enable malignant cells to be specifically targeted. These molecules can also be utilized to detect cancer early, providing clinicians with a valuable tool. Because they specifically kill cancer cells while preserving healthy ones, nanocarriers reduce the common side effects of chemotherapy [82].

## 6. Discussion

The antibacterial (AB) characteristics of metallic silver (Ag) have been recognized for a very long time. Compounds containing silver were applied in a range of treatment approaches for ailments such as ulcers, burns, and wound healing prior to the discovery that bacteria were the source of infection. This was the case both before and after the discovery of antibiotics. Ag possesses a wide variety of advantageous qualities, one of which is potent “antimicrobial” activity against a wide variety of infectious diseases [83]. In recent decades, there has been a steady increase in the prevalence of antibiotic-resistant bacteria, which presents a threat to the usefulness of these medications. As a consequence of this, the development of bactericides that are both efficient and safe is of the utmost importance [84]. The name “nano” originates from the Greek word “nano”, which can be translated to mean either “dwarf” or “very small”. The term “particles” refers to those that range in size from 1 nm to 100 nm and contain anything from 20 to 15,000 atoms of the element silver. However, the antibacterial effect is dependent on the concentration and velocity of the ionic Ag emission. Because of their nanoscale size and relatively broad exterior region, AgNPs have the potential to directly interact with bacteria, which could result in a change in the bacteria’s fundamental composition, as well as damage to the cells [85]. Synthesis can take place in a variety of ways, including physically, chemically, or biologically [86]. This yields the production of nanoparticles in a wide range of sizes and forms. It is well known that Streptococcus mutans, the primary bacterium responsible for dental cavities, is resistant to the antimicrobial and anti-adherence capabilities of AgNPs [87]. AgNPs with diameters ranging from 1 to 10 nm have been shown to possess powerful antibacterial and anti-adherence activity against the “*S. mutans* bacterium” [87,88,89]. There are many different types of bacteria, each of which has its own unique membrane structure. Gram-positive bacteria, also known as GPB, and Gram-negative bacteria, also known as GNB, have both received a significant amount of classification. The two are differentiated from one another by the thickness of the cell wall, as well as the presence of an external lipid membrane. GPB cells do not have an exterior lipid membrane; however, their cell walls are made up of a layer of peptidoglycan that is around 30 nanometers thick. GNB bacteria continue to possess a lipopolysaccharide membrane, which is encircled by a visible peptidoglycan layer that ranges in thickness from 2 to 3 nanometers [89]. The vast majority of these products are intended to eradicate “*S. mutans*” by either curative or preventative means. The vast majority of persons who were a part of our group did not receive regular updates, and the instruction regarding dental health was insufficient. Because it inhibits the enzymes that are involved in the respiratory chain and interferes with DNA synthesis [90], silver is capable of producing significant antibacterial effects when it comes into contact with a surface. Both silver diamine fluoride and silver nitrate have been shown to possess bactericidal action against the strain of *S. mutans* [36,89]. However, their usage was restricted because excessive concentrations of silver precipitate caused discoloration of the teeth. This discoloration can also induce irritation to the soft tissue around the tooth as a result of deep AgI diffusion into the dentin [91]. AgNPs are being used in biomedical sciences at a rapid pace due to their strong AB effect and high volume-to-surface ratio. [92] Research has shown that dental materials containing AgNPs exert a significant antibacterial influence. It is common knowledge that these metallic NPs produce AgI, which inhibits the ability of planktonic bacterial cells to adhere to surfaces and form biofilms. AgNPs are added to a variety of dental products, such as “acrylic resins, root canal fillings, glass ionomer cement, and pit and fissure sealants”, in order to improve the inhibition of microbial growth and the physical aspects of the modified materials [93]. These dental products include “acrylic resins, root canal fillings”, “pit and fissure sealants”, and “root canal fillings”. This is due to the fact that AGNPs possess the ability to block the colonization of microbes. Because it stops biofilm from forming on the surface, using a composite that contains AgNPs reduces the risk of microleakage and secondary caries [94,95]. This results in an improvement in the restorative material’s mechanical capabilities, which in turn helps to enhance the relationship between the dentin and the biomaterial [96,97]. AgNPs have the same efficacy as sodium hypochlorite as a canal irrigant to minimize microleakage in the root canal system [39,40,51]. Denture stomatitis is less likely to occur when these materials are used with acrylic resins because of their antifungal properties [61,77]. Osteogenic differentiation potential has the ability to produce an antibiocidal surface layer on top of titanium implants, which would help avoid peri-implantitis [41]. For decades, those working in biomedicine have been aware of the antibacterial, antifungal, and antiplasmodial actions of AgNPs [29,34,42,43,44,45,46,51,52]. 

**Figure 4:**
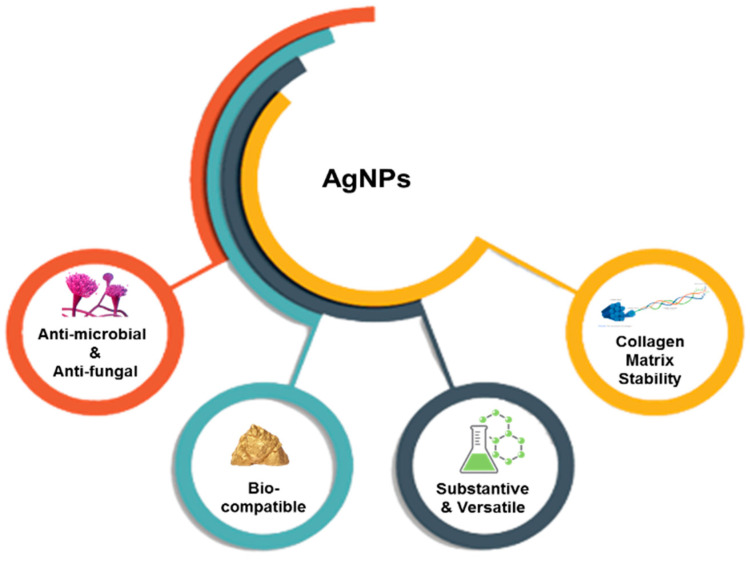
The feature scope of the silver nanoparticles.

Despite the positive findings of previous research regarding the use of silver nanoparticles (AgNPs) in dental materials, more in-depth, long-term clinical studies are necessary to evaluate their clinical efficacy and overall benefits for the dental health of patients. Because AgNPs have a greater chance of passing through cell membranes, their ability to kill bacteria is enhanced [94]. This is particularly noteworthy due to the fact that bacteria found in biofilms are far more resistant to antimicrobials than pathogens found in zooplankton [95,96,97]. The unique biological properties of AgNPs set them apart from other biomaterials that are commonly used in dentistry. As a result, AgNPs are a promising candidate for use in a wide variety of dental procedures, including those pertaining to restorative dentistry, prosthetic dentistry, endodontic dentistry, implantology, and periodontics. Nevertheless, because of the substantial antibacterial activity that silver nanoparticles (AgNPs) possess against different microbes, they have been successfully employed in a number of different fields. In the field of dentistry, AgNPs can be used for a variety of purposes, including disinfection, prophylaxis, and prevention of infections in the oral cavity. This study examines the application of silver nanoparticles in dentistry, as well as the technological advancements that are linked with the dental field. The feature scope of silver nanoparticles is shown in Figure 4.

## 7. Conclusions

In the current research, it has been demonstrated that silver nanoparticles possess specific characteristics and can be utilized in a range of dental applications. Although silver nanoparticles have greater potential in the field of dentistry, it has seen limited use in specific disciplines. In addition, it has been shown that silver nanoparticles are biocompatible with mammalian cells, further reassuring the medical community that using silver nanoparticles in dental materials is safe for humans. However, greater research is required to determine how to make the most effective use of silver complexes to assure antibacterial activity without increasing cytotoxicity throughout the various subspecialties of dentistry. This is the case despite the fact that silver complexes have been shown to be effective.

## Figures and Tables

**Table 1:** Silver nanoparticle synthesis techniques for application in dentistry.

S. No	Author	Synthesis Method	Technique Used	Merits
1	Hanif et al. [13]	Physical Synthesis	Evaporation and condensation approach and laser ablation technique	Both approaches may produce large amounts of pure AgNPs without using chemicals that damage humans and the environment.
2	Yang et al. [14]	Biosynthesis	Species such as Allium cepa, Azadirachta indica, Solanum lycopersicum	Low-cost, quick, one-step synthesis with manageable size/shape, good stability, efficacy, and security.
3	Barot et al. [15]	Chemical Synthesis	Wet chemical method	Low cost and high yield
4	Bacali et al. [16]	Physicochemical Synthesis	Variety of irradiation methods	Enormous biodiversity, environmental compatibility, scalability,

## Data Availability

Not applicable.
